# An Elusive Bullet in the Gastrointestinal Tract: A Rare Case of Bullet Embolism in the Gastrointestinal Tract and a Review of Relevant Literature

**DOI:** 10.1155/2014/689539

**Published:** 2014-01-28

**Authors:** Saptarshi Biswas, Catherine Price, Sunil Abrol

**Affiliations:** Brookdale University Hospital and Medical Center, 1 Brookdale Plaza, Brooklyn, NY 11212, USA

## Abstract

Bullet embolism within the gastrointestinal system is extremely rare. Such bullet injuries are infrequently covered in the general literature, but the surgeon should be aware of the phenomenon. Smaller caliber bullets are more common in civilian gunshot wound (GSW) events. These bullets are able to tumble through the gastrointestinal tract and cause perforation of the intestinal lumen which is small enough to be easily missed. Bullets retained in the abdominal cavity should not be dismissed as fixed and should be carefully monitored to ensure that they do not embolize within the bowel and cause occult lesions during their migration. We present a unique case wherein a bullet caused a minute perforation in the small bowel, before migrating to the distal colon, which resulted in late presentation of sepsis secondary to peritonitis.

## 1. Introduction

Bullet emboli are rare complications of gunshot injuries [1]. The relative rarity of the condition, along with potential lack of early symptoms, often leads to significant delay in diagnosis and treatment of the problem. Consequences of such a missed injury can be devastating and prove fatal as shown in our case. The easy availability of guns in the United States, complimented by the upsurge of gun violence in a civilian urban setting, has increased the possibility of encountering bullet embolism [2].

The majority of such emboli are notoriously asymptomatic and thus can be missed on initial evaluation. Whenever initial workup fails to visualize the bullet or the entry and exit wounds do not match up, a need for detailed interrogation should be triggered in the mind of the clinician. Unexplained trajectories of bullets should raise the suspicion of bullet embolism.

In this paper, we describe a rare case of gastrointestinal bullet embolism where a small-caliber bullet perforated the small bowel, migrated distally, and eventually lodged in the distal colon, which resulted in a missed injury, leading to sepsis and the demise of the patient.

## 2. Case Report

An 18-year-old male was brought into an urban, level-one trauma center by emergency medical service after reportedly being shot in the buttocks. On arrival, the patient was awake, alert, and oriented to person, place, and time. Airway and breathing were determined to be patent and adequate. The patient's initial vitals were T97.5°F, BP109/75, HR103, and RR20. Venous access was secured via placement of two large-bore intravenous catheters and insertion of a femoral vein triple lumen catheter. Patient was moving all four extremities upon arrival.

**Figure 1:**
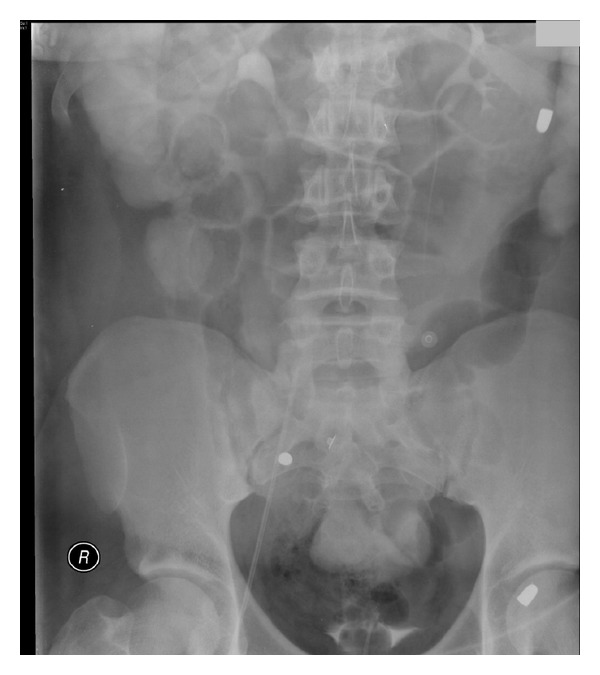
Note the bullet in the left upper quadrant of the abdomen.

**Figure 2:**
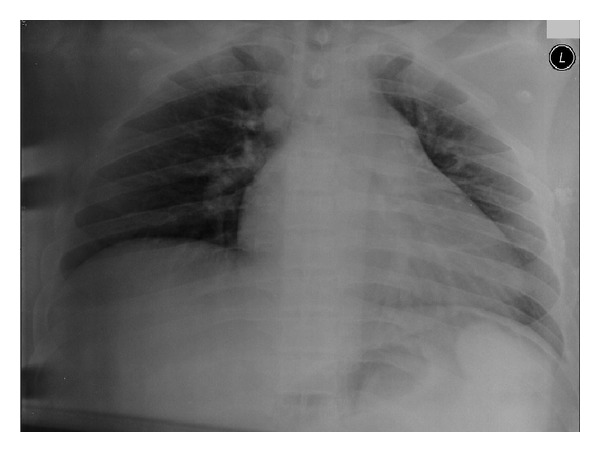
The initial chest X-ray on presentation to the emergency department shows no pathology.

All clothes were removed and, while stabilizing the cervical spine, the patient was rolled and evaluated for injuries on all surfaces. Noted GSWs included one near the coccyx, two in the left lateral gluteal region, one in the right lateral gluteal region, and one in the left upper medial thigh. A secondary survey revealed a soft, nondistended abdomen with active bowel sounds and a rectum with good tone and no bleeding on the examining finger. Patient's initial laboratory values were WBC 9.9, hemoglobin 14, hematocrit 41.7, platelets 241, sodium 141, potassium 4.3, chloride 101, bicarbonate 28, BUN 14, creatinine 0.9, and glucose 127. X-rays of the chest, abdomen, and pelvis were taken (Figures 1 and 2).

**Figure 3:**
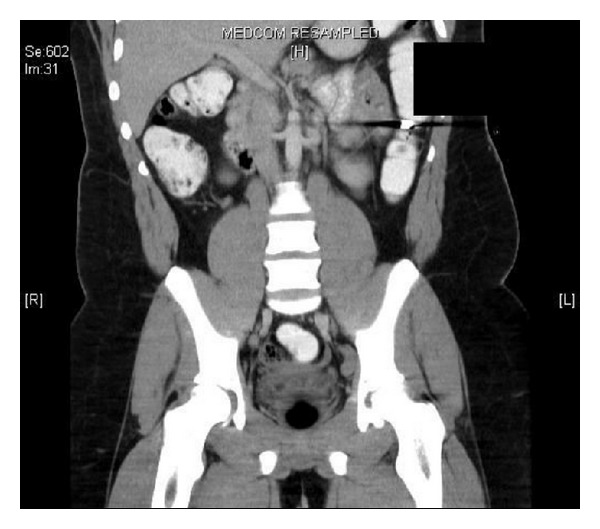
A CT reveals a bullet in the lumen of the small intestine in the right upper quadrant.

A CT scan with triple contrast enhancement of the chest, abdomen, and pelvis was obtained. The CT revealed a bullet in the left upper quadrant of the abdomen, a bullet in the right hemisacrum, and a bullet lodged in the posterior column of the left acetabulum. There were air bubbles in the peritoneum, which could be secondary to entrance of the bullet into the peritoneal cavity or secondary to intestinal injury. There was a minute amount of free fluid in the pelvis. There was no evidence of colonic or vascular contrast extravasation (Figure 3).

The patient was taken to the operating room for an exploratory laparotomy to rule out intra-abdominal hollow viscus or intraperitoneal injury. The small bowel, from the ligament of Treitz to the ileocecal valve, was evaluated independently by two surgeons. The large colon was mobilized starting with the left colon down to the sigmoid colon, then the transverse colon, and finally the right colon. Intraoperatively, neither gross bowel nor intraperitoneal injuries were noted. The patient was transferred to the postanesthesia care unit in stable condition.

**Figure 4:**
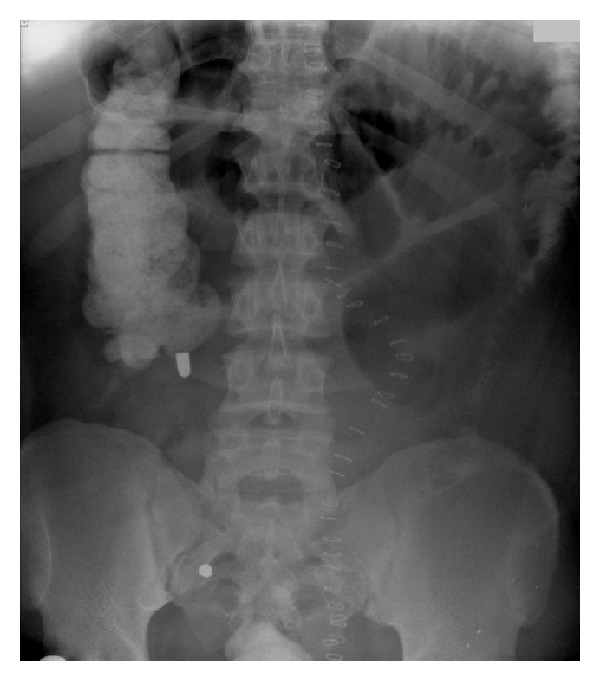
Note that the right upper quadrant bullet has now embolized to the left lower quadrant of the abdomen.

The patient's postoperative course was uneventful with the exception of vomiting and an associated WBC count rise from 7.9 to 11.3, on post operative day 3. An abdominal X-ray was performed to determine small bowel ileus versus obstruction. A small, rounded metallic density was again noted overlying the right sacrum. The bullet previously seen overlying the left upper quadrant was no longer visualized at that site. There was a similar-appearing bullet seen in the right abdomen at the level of L4, possibly just inferior to the right colon. It is possible this bullet was within the small bowel. If it were, this would explain its transit. If it was in the bowel, it may have been lodged at the ileocecal valve. There was also associated small bowel dilatation with air-fluid levels seen. Small bowel obstruction could not be excluded and, theoretically, might be attributed to the bullet. It was also possible the bullet may have moved due to the exploratory laparotomy. There was retained contrast material noted in the colon and rectum, which were not significantly dilated. If there was a small bowel obstruction it was either incomplete or early (Figure 4).

On POD 4, the patient's WBC count increased to 19 and he spiked a fever of 38.4°C. Cultures of blood, urine, and sputum were obtained. No bacterial growth was ever realized from these cultures. A chest X-ray was obtained and deemed to be unremarkable. On POD 5, the patient was afebrile for a period greater than 24 hours, ambulating, and tolerating diet. He had stable vitals, a normal WBC count, and normal bowel function; therefore a decision was made to discharge the patient to his home.

The patient presented to the emergency room on POD 8 with hypotension and abdominal pain since that morning, which was accompanied by coffee ground emesis. Physical examination revealed a jaundiced male who was awake, alert, and oriented to person, place, and time but in obvious discomfort. His abdomen was distended with positive signs for peritonitis. Labs obtained at this time were WBC 24.2, hemoglobin 12.4, hematocrit 37, platelets 496, sodium 131, potassium 6.1, chloride 94, bicarbonate 15, BUN 30, creatinine 3.4, glucose 124, ALT 150, AST 200, total bilirubin 2.2, creatine kinase 203, lactate 7.18, ammonia 180, INR 1.3, PT 14.7, and PTT 23.7. Arterial blood gas measurements (pH 7.27/pCO_2_ 33.7/pO_2_ 96.1/HCO_3_ 15/O_2_ sat 96%/base deficit −10.9) showed significant metabolic acidosis. Volume resuscitation was started, and an NGT and Foley catheter were placed.

**Figure 5:**
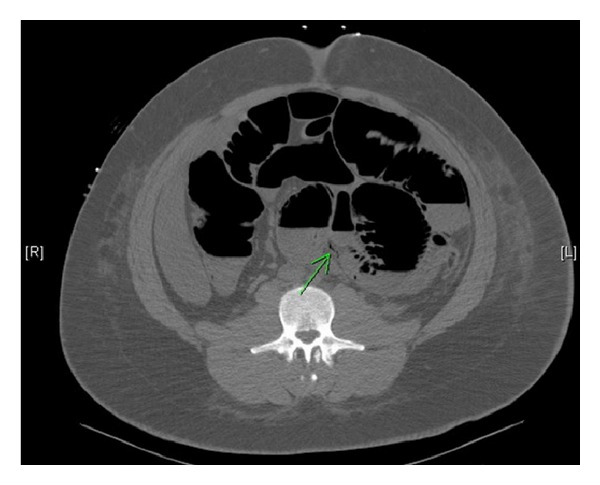
Note the pneumatosis intestinalis now present.

**Figure 6:**
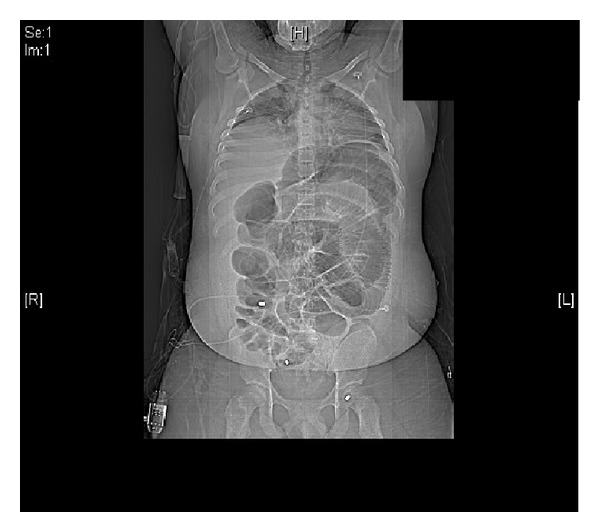
Note the extensive lung and bowel pathology. The bullet in question is still lodged in the left lower quadrant.

A CT of the chest, abdomen, and pelvis showed pneumatosis intestinalis (Figure 5) of the small bowel. Dilated loops of the small bowel in the right mid-abdomen had peripheral curvilinear air, which was consistent with air in the bowel wall and was due to pneumatosis, probably due to ischemia. In addition, there were tiny bubbles of air between the dilated small bowel loops which was most likely air in the blood vessels or extraluminal air. Jejunal loops were markedly distended and contained air and fluid without wall thickening. These changes are more consistent with proximal small bowel obstruction than with ileus. The cecum and proximal ascending colon contained air and fluid and were normal sized. The hepatic flexure, transverse colon, and splenic flexure were moderately dilated and contained air. The descending colon was totally collapsed. The sigmoid colon was collapsed and contained minimal air. There was a large amount of pelvic and right upper quadrant ascites, which was increased in size in comparison to the last CT. There was a bullet located in the right lower quadrant, perhaps in the small bowel loops, which was previously in the left upper quadrant. A metallic bullet fragment was on the right side of the sacrum and posterior column of the left acetabulum (Figures 5 and 6). There was a moderate amount of fluid in the lower abdomen and right upper quadrant, which was partly loculated and could represent infected fluid.

After CT, the patient started having bilious vomiting, and his blood pressure plummeted to 99/66. A right internal jugular triple lumen catheter was placed and normal saline boluses were begun. The patient was intubated and received IV cefoxitin and piperacillin/tazobactam for empiric polymicrobial coverage.

The patient was taken to the operating room. Coffee ground-appearing emesis was now present in the suction canister of the NGT. Urine output was scant. Intraoperatively, ischemic bowel was noted. The small bowel was resected from an area distal to the ligament of Treitz to the distal ileum, and the remaining portion was anastomosed. A portion of the large bowel proximal to the splenic flexure was resected with the proximal portion brought to the skin to form a colostomy.

Intraoperatively, the patient received 8 L of normal saline, four units of packed red blood cells, and two units of fresh-frozen plasma. The patient received four additional units of fresh-frozen plasma postoperatively. The patient lost vital signs 4 hours after operation. An attempt was made at resuscitation, as per advanced cardiac life support guidelines; however, the patient expired.

An autopsy report was obtained. The cause of death was determined to be intestinal necrosis and peritonitis due to multiple gunshot wounds of the buttocks with perforation of the intestine. There were four penetrating gunshot wounds of the buttocks. One of these caused perforation of the peritoneal cavity and intestine, which lead to marked peritonitis and resultant intestinal necrosis. The collective wound tracks of the penetrating GSWs passed throughout the soft tissue of the buttocks in a back-to-front direction. Only one wound track entered the peritoneal cavity. This track ended in the lumen of the intestine. It was associated with marked yellow-red fibrinous exudates, which surrounded the right liver, spleen, mesothelial surfaces of the peritoneal cavity, and patches of the remaining serosal surfaces of the small and large intestines.

Three bullets were recovered postmortem. A grey-discolored, small-caliber bullet was recovered from the intraperitoneal track. A small-caliber, yellow, metal-jacketed bullet was recovered from the left pelvic bone at the sacroiliac joint. A similar-appearing bullet was recovered from the anteromedial right thigh.

The pleural cavities each contained approximately 200 mL of clear yellow fluid and were free of adhesion. The stomach contained 150 mL of dark red liquid and had a red mucosa. The remaining intestines had alternating sections of smooth, tan, edematous mucosa with areas of dusky red discoloration. The colon wall was markedly edematous and without perforating defects. There were moderate adhesions between the bowel loops. Lung microscopy revealed moderate acute bronchioloalveolar pneumonia. The liver was found to have centrilobular ischemia. The serosal surface of the small and large intestines showed mixed chronic and acute inflammatory infiltration. The muscularis layers of the small intestine had hemorrhagic necrosis with focal preservation of the overlying mucosal epithelium.

## 3. Discussion

Embolism by definition refers to the migration of solid, liquid, or gaseous substance from its point of origin to a distant site. The solid particle can be missile, thrombus, or even a medical device. The liquid can be amniotic fluid. The gaseous substance could be a nitrogen or atmospheric air bubble [3, 4]. Emboli are propelled through a vessel by the flow of blood, air pressure, or active or passive body movements [5].

The first bullet embolism was reported by Thomas Davis in 1834. This case was subsequently cited later, in 1919, by Bland-Sutton [6]. Most of the reports published in English literature are in the form of isolated case reports. One of the earliest and largest report series was by Mattox et al. in 1979. It comprised 28 cases spanning 12 years and provided one of the most significant contributions to understand this rare phenomenon [7, 8].

Krispin et al. [3] has described trauma caused by ballistics as perforating and penetrating. In case of penetrating trauma, the bullet loses its kinetic energy during its travel along its trajectory and remains within the domains of the body cavity. Bullet embolism occurs when a bullet penetrates the body, its movement is stalled, and then it is carried away from its initial site of lodgement to a distant location.

Bullet emboli, in themselves, are rare phenomena. Most reports are regarding military conflicts or vascular emboli. A study by Rich et al. [9] of 7,500 GSWs during the Vietnam War revealed only 22 cases complicated by foreign body emboli (0.3%). This study shows, in a battlefield environment, the incidence of bullet embolism was exceedingly low. Only 3 cases described a whole bullet vessel embolus. The other cases described mostly fragments from explosive devices.

A 10-year retrospective review by Sandler et al. [10] revealed only 46 cases of GSW that resulted in missile emboli. In 1996, Adegboyega et al. [11] reported only 160 cases of bullet emboli since 1834. Michelassi et al. [12] reviewed 153 cases of bullet emboli that were reported in English literature and were mostly amongst the civilian population. The authors concluded that embolization occurred twice more frequently in the arterial system compared to the venous system. They reported 15.5% occurrence when the aorta was the source of the arterial bullet emboli. The left leg was twice more likely than the right to be involved, which can be explained by the asymmetry of the aortic bifurcation. Twenty percent of these patients with arterial bullet embolism were asymptomatic, while 66.7% had peripheral ischemia. More than 70% of missiles that penetrate into arterial circulation enter via the thoracic or abdominal aorta, according to Slobodan et al. [13]. In odd events, missile emboli have even been reported to enter arterial circulation through the heart [12–17].

Isolated reports of distal arterial embolization from peripheral arteries have also been published [18, 19]. The embolism is anterograde in the majority of cases. Rare cases of retrograde (venovenous) [20–22] and paradoxical [23] emboli have also been reported. Schmelzer et al. [22] and Rich et al. [9] mentioned that arterial missile emboli outnumber venous ones by 4 : 1. Biswas et al. [2] described a unique case in which the autopsy study revealed the bullet entered through the thoracic aorta, flowed through the systemic circulation, and eventually lodged into the right popliteal artery. The embolism in this case might have occurred shortly after the initial injury or in the perioperative resuscitative phase. Other reports have shown that embolization has been delayed for days, weeks, or longer [8, 24–26]. Wales et al. [27] have reported a unique case of a right ventricular bullet embolism that manifested clinically 4 years after initial insult. Cardiac bullet emboli can cause cardiac irritability, delayed embolism [22, 28, 29], and recurrent pericardial effusions and may even interfere with valvular functioning [7].

Clinical manifestations are often attributed to the site of penetration of the vascular system, the injury to other viscera, or the site of the embolization. These can all cause delay in diagnosis, which can result in adverse outcome or even death. The signs and symptoms may not correspond with those expected from the presumed course of the bullet. When a bullet is found in an unexpected location, deviant from the conventional trajectory, embolism should be suspected. Pain, claudication, paresthesia, gangrene, pleural or pericardial effusion, endocarditis, sepsis, cardiac arrhythmias, pseudoaneurysm, or even neuroses and psychoses are some of the delayed presentations [7].

Although the final endpoint of an embolized bullet is unpredictable, some consistent patterns have become elucidated as more cases are reported. A peripheral venous embolus generally terminates in the right side of the heart or in the pulmonary artery. A bullet that enters the right side of the heart will commonly embolize to the pulmonary artery, while pellets or small missiles starting from the left side of the heart are likely to embolize into the middle cerebral arteries. Kase et al. concluded that the right brain is affected more often (70%) than the left [28].

The flow pattern into the innominate artery, which is the first and largest branch of the aortic arch, is the causative factor. Bullets from the thoracic or abdominal aorta usually lodge into the arterial tree of the lower extremity. Left leg involvement is more likely due to the axis of the left common iliac artery forming a narrower angle (30 degrees) from the aorta than from the right side (45 degrees) [19].

The guns and ammunition used in a trauma are also important to identify, because bullet emboli are more common with smaller, blunt-nosed, short-length, or low-velocity bullets [1]. Patel et al. [8] have concluded that the low incidence of bullet embolism is because two major prerequisites need to be satisfied for a projectile to become an embolus. First, the bullet should have little kinetic energy remaining at the precise instant it enters the blood vessel. Second, the diameter of the bullet must be less than the diameter of the blood vessel it penetrates. Patel et al. [8] reported an embolus incidence of 55% with a 0.22-caliber handgun and 27% with a shotgun. In a review by DiMaio and DiMaio, in 24 cases of bullet embolism where the caliber or type of the gun was known, a small, 0.22-caliber bullet accounted for the majority of emboli [29]. Embolism is rarely caused by high velocity bullets, as evidenced by its low incidence in war literature [30].

Small-caliber bullets are more prone to tumbling, may not pierce blood vessels or intestinal lumens, have a slower velocity, and are able to fit through peripheral blood vessels that are too narrow for large-caliber bullets. These small-caliber bullets possess low kinetic energy at the time of impact. The primary ballistic feature of the short 6.35 mm caliber missile is its small weight (3.2 g) and low initial velocity (240 m/s), which translates into low initial kinetic energy (92 J). Therefore the penetrating power of such a missile is limited. Such bullets are blunt-nosed and thus are less streamlined. They are more retarded by tissues and subsequently lose a greater amount of kinetic energy after penetration into the body. The degree of bluntness of the nose also determines the initial value of the area of interphase between the bullet and the tissue and thus the drag of the bullet. Wobbling and tumbling of a bullet during its passing through the tissues cause even further loss of kinetic energy [13].

The density, elasticity, and strength of the tissue hit by a bullet, as well as the length of the wound tract, can influence the loss of initial kinetic energy, as in our case. The denser the tissue the bullet has to overcome is, the greater the retardation is. This, subsequently, leads to greater loss of kinetic energy. Increased density also shortens the period of gyration, which eventually results in greater retardation and increased energy loss [13].

As discussed earlier, the increase in gun violence in the civilian setting has resulted in more reports of bullet emboli. Most of these cases describe embolism in larger vessels, obstruction of distal systemic vascular tree, or even pulmonary or paradoxical emboli. Shiver et al. describe a case of urethral obstruction due to the passage of a projectile retained in the genitourinary system [31]. Raz et al. [32] and Bozeman and Mesri [33] present cases describing acute urinary retention following late migration of a retained bullet. Smalls and Siram describe a unique case of a wandering bullet in which the bullet caused esophageal injury and lodged in the stomach [34]. DiMaio [35], in an article about the application of pathology to crime investigation, has cited a case of bullet migration from the stomach to the intestines. DiMaio has described two interesting cases of bullets expelled through the oral orifice.

In the first case, a spent bullet from an entry point in the back was recovered from the oral cavity, while the second described a missile from a chest wound that halted within the lungs and was coughed up by the victim [34]. One must also consider that “lost” bullets may have actually passed uneventfully through the gastrointestinal tract, as reported by Morrow et al. [36].

A bullet becoming lodged in the intestine is an extremely rare event even though bullet injuries constitute the majority of perforating abdominal trauma and a large part of penetrating trauma [3, 37–39]. Although rarely reported, gastrointestinal embolization should be taken into consideration when searching for a missile in the abdomen during an exploratory laparotomy. In a case in which a bullet halts within the intestines, lack of awareness of the spatial location may result in futile seeking of the missile in the abdominal cavity.

Sometimes a penetrating bullet that punctures the abdominal cavity has enough kinetic energy to perforate the intestines and stop after striking the vertebral column, abdominal musculature, or even just beneath the skin [3]. However, in some rare cases of low-velocity ammunition, the bullet course ends within hollow viscus such as the small or large intestine. Such cases are extremely rare and seldom reported.

In the present case, the bullet had penetrated the small bowel through a small laceration and moved from its initial location as evident from the initial CT scan to a different location seen in subsequent imaging. The migration can be attributed to a peristalsis or even palpation and manipulation of the bowel during the surgical exploration. The bullet was never expelled from the body by defecation, probably due to decreased bowel movement as a component of the ileus and peritonitis that followed the undetected perforation.

Along with low-velocity, small-caliber bullets, bullet fragments or pellets are also prone to embolization. This explains the scenario in our case where a 0.22-caliber was fired probably from a distance of 10–20 feet, which resulted in kinetic energy sufficient to penetrate the intestinal lumen but not enough to exit it. The fact that the patient was morbidly obese and the entry site was the gluteal region slowed the kinetic energy of the projectile. The bullet was also small enough to cause an intestinal entry wound and minute enough to subsequently become sealed enough to be missed on open exploration. The bullet's movement compounded the initial problem of missed location.

Therefore, in rare instances where a bullet is not immediately located, it is important to determine the initial bullet trajectory before its path was possibly changed in the body. To determine this, first the relation of the location of the victim to the shooter must be determined. Thereafter, the axis of the gun barrel must be ascertained. Factors postulated to be responsible for determining distant lodgment sites are (I) power of the missile, (II) caliber and shape, (III) site of penetration into the vascular bed, (IV) effect of gravity especially in the low-pressure venous system, (V) respiratory activity, (VI) force of blood flow, (VII) position of the victim at the moment of penetration of the embolus into the circulation, and (VIII) relative size and angle of the arterial branches [5, 7, 40, 41].

All projectiles can potentially carry bacteria into the wound. In addition they can cause introduction of skin flora, clothing, and foreign particles, any of which can act as a nidus of infection independently [42]. A unique case of *Clostridium difficile* subacute bacterial endocarditis was reported by Bilsker et al. [43]. It was attributed to a bullet that penetrated the left ventricle and subsequently the right subclavian artery.

The argument for bullet extraction after bowel injury includes a decreased risk of sepsis. Sarmiento et al. [44] found that after abdominal GSW, if colon wounds occurred and the bullet was retained, the incidence of shock was higher than if the bullet was removed and surrounding soft tissue debrided. It was also found that if the missile entered the colon but exited spontaneously (in this study the exit was not via embolism in any of the cases, but rather through-and-through penetration), the resultant internal tract should be subjected to a thorough washout and removal of damaged tissue.

Another consideration, which Sari et al. [45] discuss, is elevated blood lead from gunshot injuries that result in ingestion. It was found that the gastrointestinal tract is efficient in absorbing lead from retained bullets. In these instances, there is rapid elevation of blood lead immediately after injury. This level persists even three years after incident.

A very high index of suspicion is of utmost importance to identify a missile embolus. Accounting for all bullets and all bullet wounds allows for a basic reconstruction of each bullet's trajectory, thus identifying organs and tissues at risk of damage. If the number of entry wounds does not equal the number of exit wounds or the clinical signs or symptoms and radiologic imaging do not correlate with the injury, the possibility of bullet embolism should be entertained. Unexplained clinical findings should also trigger the suspicion of missile embolism.

Although the bullet rule is a helpful concept, there are well-documented exceptions to the rule. Two bullets fired in unison as tandem bullets from a handgun entering the body through the same entrance wound, but leaving two bullets in situ, although exceedingly unusual, have been documented [46]. Another example is a bullet that ricochets within the calvarium, entering and exiting from the same point [47].

## 4. Conclusion

Migratory intraluminal bullets may produce complex and confusing scenarios in patients with GSWs, often resulting in diagnostic and therapeutic dilemmas. This case is a glowing example how a bullet embolism initially unaccounted for eventually influenced the course and outcome of a trauma patient. Given the grave outcome of a missed small bowel injury, an additional confounding variable such as an intraluminal bullet migration presents a truly formidable challenge for even the most experienced trauma surgeon.

This case emphasizes that the true path of a bullet or fragment cannot be always ascertained based on the site of the entrance wound. However, when the suspicion of a bullet embolism has been raised, the missing projectile should be thoroughly accounted for by a thorough and meticulous search and the resultant damage addressed even if the patient is asymptomatic on initial presentation. The real purpose of the bullet rule is to remind clinicians to look for additional injuries and to be diligent in the evaluation of patients who have suffered gunshot wounds.

The consequence of a missed bullet embolism may prove fatal, as our case illustrates.
